# Supplementary material for: High Accuracy Indicators of Androgen Suppression Therapy Failure for Prostate Cancer—A Modeling Study
Source: Cancers (Basel). 2022 Aug 20;14(16):4033. doi: 10.3390/cancers14164033 (PMC9406554; doi:10.3390/cancers14164033)
Supplement: Supplementary file 1 [file cancers-14-04033-s001.zip › cancers-1841805-supplementary.pdf]

Supplemental Material

Model Formulation:

The model we present was formulated incrementally as a series of changes, modifications, and occasional failures over time. This is a summary of the steps taken to get from an established starting point to the final form of the model presented in this publication.

We began with a model published by Baez et al (1). The Baez model is a system of four differential equations that represent: the volumes of two cancer cell subpopulations, the cell quota of androgen, and the serum concentration of PSA. The model is as follows:

$$\frac{dx_1}{dt} = \mu\left(1 - \frac{q_1}{Q}\right)x_1 - \left(d_1\frac{R_1}{Q + R_1} + \delta_1 x_1\right)x_1 - \lambda x_1$$
$$\frac{dx_2}{dt} = \mu\left(1 - \frac{q_2}{Q}\right)x_2 - \left(d_2\frac{R_2}{Q + R_2} + \delta_2 x_2\right)x_2 + \lambda x_1$$
$$\frac{dQ}{dt} = (\gamma_1 u(t) + \gamma_2)(Q_{max} - Q) - \mu\left(\frac{(Q - q_1)x_1 + (Q - q_2)x_2}{x_1 + x_2}\right)$$
$$\frac{dP}{dt} = bQ + \sigma(Qx_1 + Qx_2) - \epsilon P$$

When we reproduced the Baez model, we found that we could rarely recreate the whole range of androgen and PSA data. Peaks of data caused by spikes in data values were often truncated to some lower level. Our original goal was to discover if adjustments could be made that would allow the model to better represent these peaks.

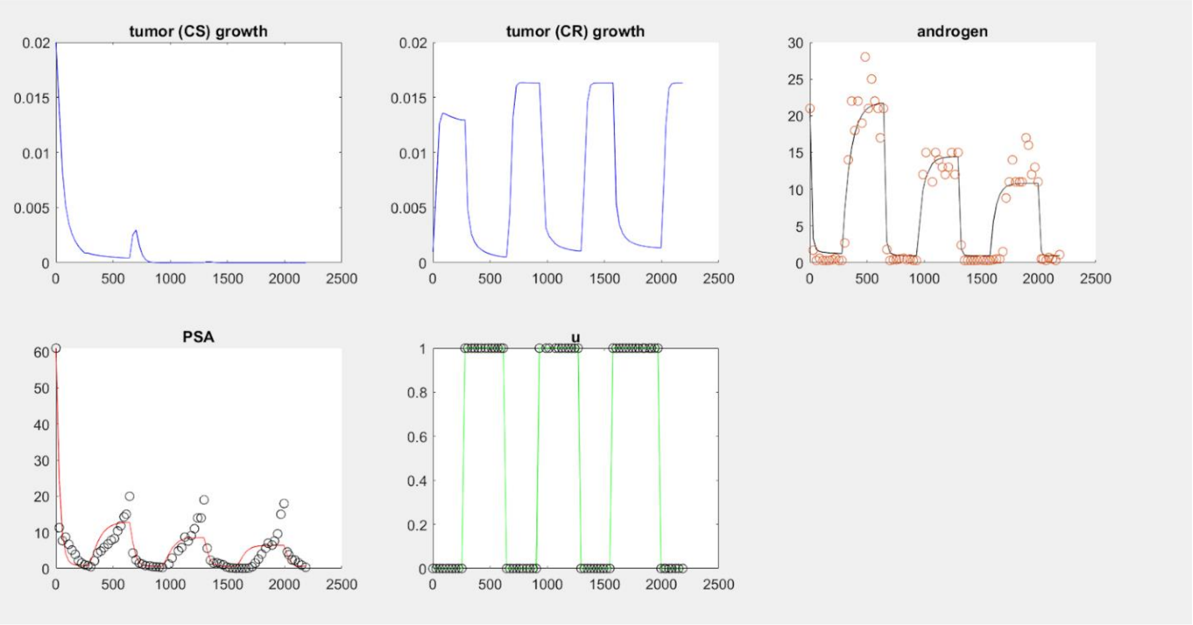

Figure S1: This one result of our recreation of the Baez model. The data are represented by black circles, while the simulated results are solid lines. The figure titled ‘u’ shows when the patient was

on or off treatment. This purpose of this figure is to show that, in the case of both androgen and PSA, the model was not reproducing the entire range of data. The upper end of measurements, or 'peaks', are cut off.

To that end, our first change was to condense the two death rate parameters into just one term. When we examined the optimized parameters returned to us by the fmincon function, we noticed that there was always very little difference between the values of d_1 and d_2. We decided that the difference was small enough to justify merging these terms so that the model might better identify the other parameters. On the other hand, we also split the σ parameter into σ_1 and σ_2. We did this to allow for the possibility that both subclones were producing PSA at different rates. This is what the model looked like after making those changes.

$$\frac{dx_1}{dt} = \mu\left(1 - \frac{q_1}{Q}\right)x_1 - \left(d\frac{R_1}{Q + R_1} + \delta_1 x_1\right)x_1 - \lambda x_1$$
$$\frac{dx_2}{dt} = \mu\left(1 - \frac{q_2}{Q}\right)x_2 - \left(d\frac{R_2}{Q + R_2} + \delta_2 x_2\right)x_2 + \lambda x_1$$
$$\frac{dQ}{dt} = (\gamma_1 u(t) + \gamma_2)(Q_{max} - Q) - \mu\left(\frac{(Q - q_1)x_1 + (Q - q_2)x_2}{x_1 + x_2}\right)$$
$$\frac{dP}{dt} = bQ + (\sigma_1 x_1 + \sigma_2 x_2)Q - \epsilon P$$

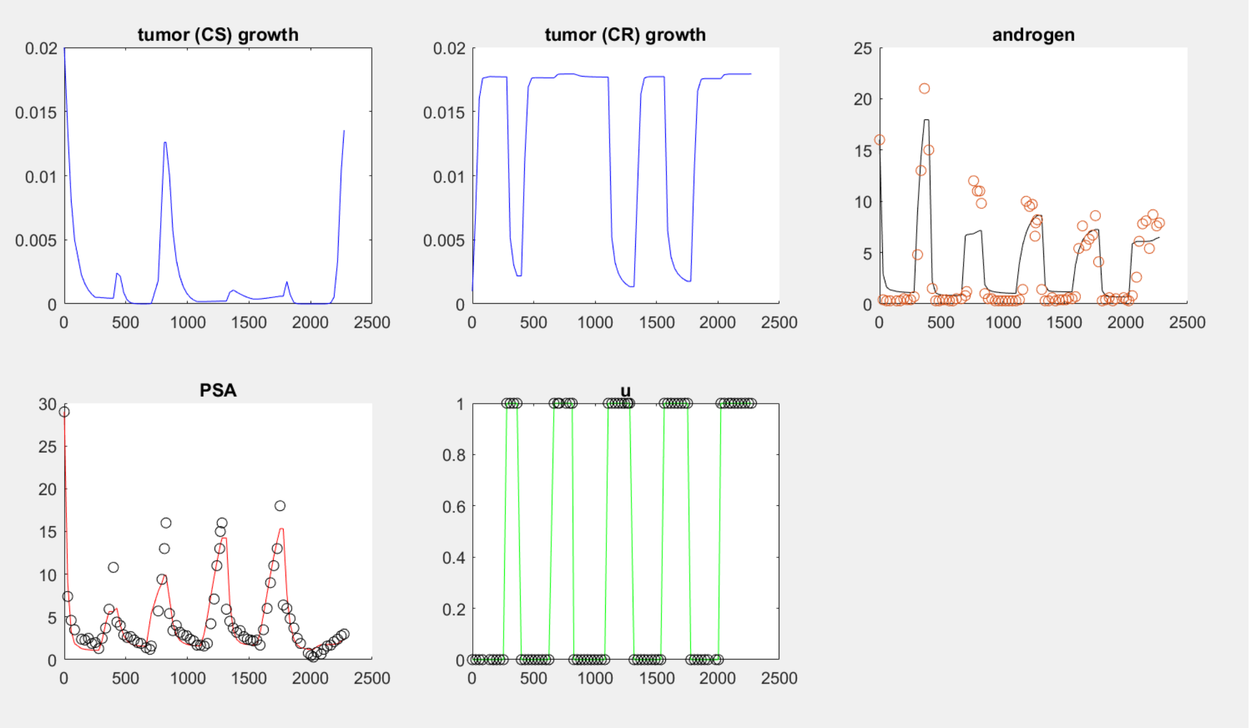

Figure S2: An example of the results produced by the first series of modifications to the Baez model. The two death terms, d_1 and d_2, were merged into one, d, and the σ parameter was split into σ_1 and σ_2. The apparent result of these two adjustments was an improved capacity for the model to represent the data.

Taken together, these two changes were found to somewhat improve the model's ability to simulate larger measurements. An example of the results may be seen in Figure S2. These changes were tested separately, and it was found that making the change to σ alone was not sufficient to apparently improve the result.

Next, we implemented three substantial modifications. These changes were tentative and experimental, so they were all tested separately and in combination with each other. In addition, all of these model variations were tested with and without the addition of a degradation term to the dQ equation.

The first modification was the complete removal of the death terms. Negative growth is already made possible by the Droop equations in the growth terms (growth is negative when $Q < q_{\mathrm{i}}$), and we wanted to see if this negative pressure would be sufficient to control the cell populations without the death terms.

The second modification was the removal of the dx_2 equation. We wanted to know exactly how much the second layer of complexity contributed to the outcome. We thought it possible, since we were continually fitting the parameter q, that resistance might be adequately modeled by the dynamics of q alone. If we could remove one of the differential equations without substantially damaging the result, it would make it much easier for fmincon to identify the remaining critical parameters (see section below for more information on fitting and critical parameters).

The third modification was a substantial change to the PSA equation and involved incorporating the Droop functions from the growth terms into the PSA production terms. This is a novel modification that introduces an entirely different interpretation of what motivates PSA production and is discussed in the main body of this publication.

This is the system of differential equations that incorporates all three of these modifications, and includes the degradation term in the dQ equation:

$$\frac{dx}{dt} = \mu\left(1 - \frac{q}{Q}\right)$$
$$\frac{dQ}{dt} = \gamma(t)(Q_{max} - Q) - \mu(Q - q) - \delta Q$$
$$\frac{dP}{dt} = \mathrm{bQ} + \sigma\left(1 - \frac{q}{Q}\right) - \epsilon\mathrm{P}$$

The products of this system were surprising, but still useful, and may be seen in Figure S3. Clearly this is a failed result, but it was immediately apparent where we had made the mistake: by applying the Droop functions to the dP equation, we had conferred the possibility for negative growth (due to a lack of androgen) to PSA as well. Furthermore, we can see that by removing the death terms that the tumor volumes and PSA concentrations have both grown far beyond the limits of believability.

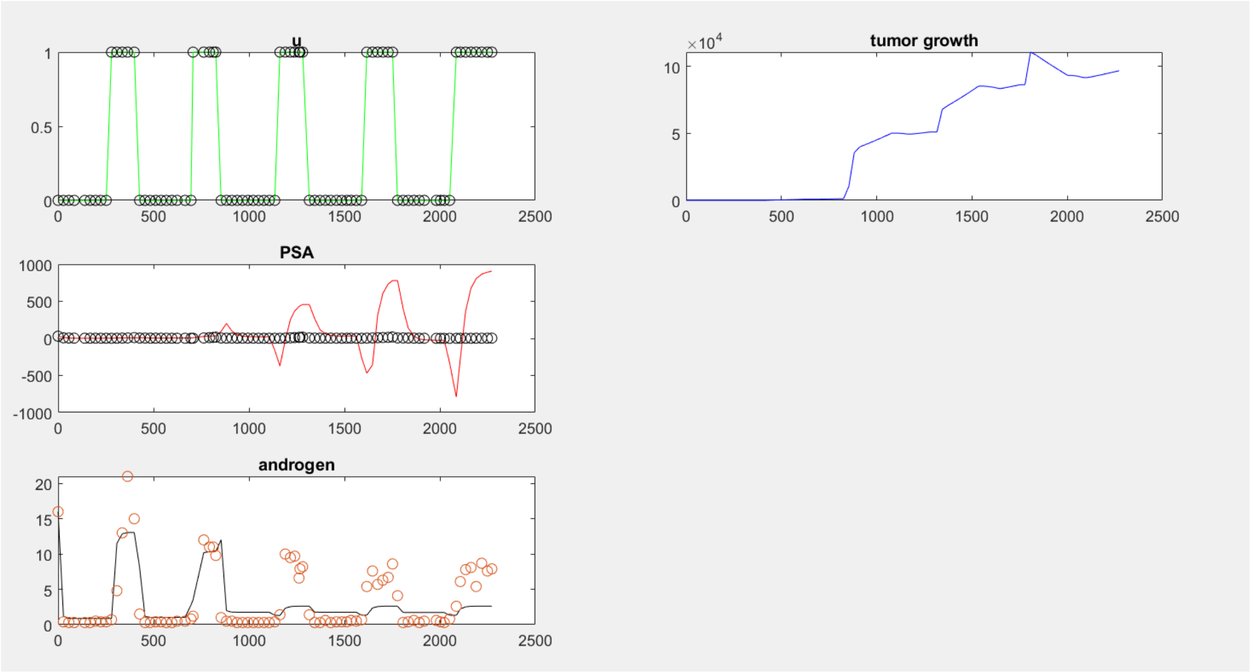

Figure S3: This figure shows the result of removing the death terms and applying Droop functions to PSA production. This is clearly a failed attempt. PSA oscillates between positive and negative values, which is clearly impossible. Furthermore, by the end of treatment the magnitude of the tumor volume is entirely incredulous.

To rectify this mistake, we applied maximum functions to all the related Droop terms. Negative growth needed to be eliminated from PSA production, but for consistency we applied the maximum functions to the growth terms as well. In doing so, we eliminated the built-in capacity for negative growth in the *dx* equations, where it was needed. This, in addition to the explosive, runaway growth of the cell volumes seen the previous result, required us to reintroduce death terms to the model.

We initially tested two variations on these death terms, $d_1 x_1$ vs $d_1 x_1^2$, and found that the latter was necessary to keep the populations under control. When we reintroduced these death terms we used two separate death rate parameters: d_1 and d_2.

After experimenting with all the variations, and implementing these corrections, the best performing model was this (see also Figure S4):

$$\frac{dx_1}{dt} = max\left\{\mu\left(1-\frac{q_1}{Q}\right)x_1, 0\right\} - d_1 x_1^2$$
$$\frac{dx_2}{dt} = max\left\{\mu\left(1-\frac{q_2}{Q}\right)x_2, 0\right\} - d_2 x_2^2$$
$$\frac{dQ}{dt} = \gamma(t)(Q_{max} - Q) - \mu\left(\frac{(Q-q_1)x_1 + (Q-q_2)x_2}{x_1 + x_2}\right) - \delta Q$$
$$\frac{dP}{dt} = bQ + max\left\{\sigma_1\left(1-\frac{q_1}{Q}\right)x_{1,}0\right\} + max\left\{\sigma_2\left(1-\frac{q_2}{Q}\right)x_{2,}0\right\} - \epsilon P$$

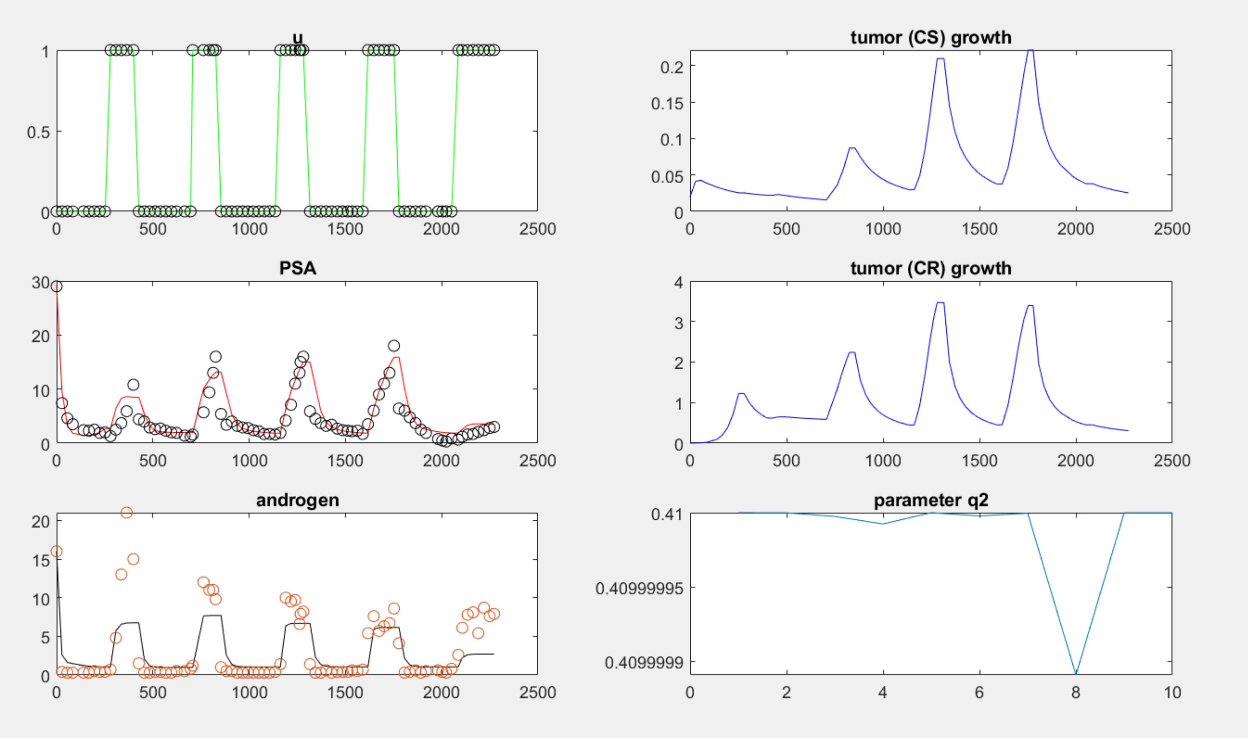

Figure S4: This figure is an example of the result produced by the model when the Droop functions were applied to the PSA production terms and upon reintroducing death terms to the growth equations. This result was viewed as a success, but we identified a remaining challenge in that the volume of the resistant cell colony was still unrealistically large.

Our next challenge was that the simulated tumor volumes were often impossibly large. In Figure S4, for example, we see that the volume of the treatment-resistant subpopulation is approaching four liters. Our next task was to implement changes that might affect a reduction in those numbers.

We first tested a range of new values for the death rate parameters, with varying levels of success, before again merging them into a single parameter *d*. We also tested a range of new values for the growth parameter μ. Unable to find the bounds for μ that gave the desired result, we incorporated a change in how the μ parameter was optimized. Whereas before we programmed fmincon to optimize μ, alongside other parameters, against a short, two-cycle test segment, the program was changed such that μ was fitted first, before any other parameters, against an even shorter, half-cycle segment. The resulting value of μ was then fixed before any other parameters were optimized. This was found to successfully reduce the upper bounds of our tumor volumes without substantially changing the underlying dynamics.

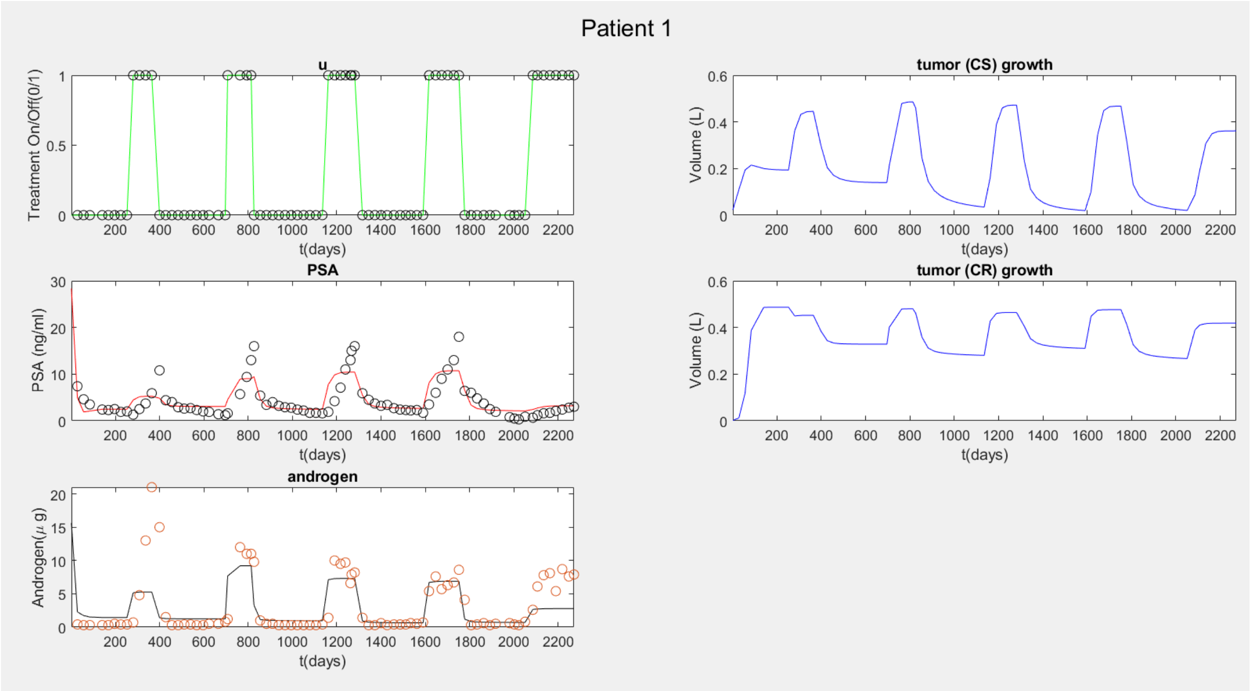

Figure S5: Changing the method of optimizing the single parameter μ resulted in a more realistic range of resistant tumor volume. Notable is that the CS and CR populations co-exist throughout the course of treatment, with one never overtaking the other.

Feeling that the volumes of x_1 and x_2 were now under control, we moved on to something new. We noticed that the resistant and susceptible tumor populations, x_1 and x_2, often coexisted together throughout the course of the treatment. This can be seen in Figure S5 where, although both populations grow and shrink according to treatment status, one is never seen to outcompete the other. This did not conform to our expectations, which were for the resistant subpopulation to become dominant as treatment is applied repeatedly over time. To address this, we decided to modify the death terms once again, this time to introduce an element of interspecific competition between these two subpopulations.

These are the first two differential equations after the introduction of interspecific competition:

$$\frac{dx_1}{dt} = max\left\{\mu\left(1 - \frac{q_1}{Q}\right)x_{1,}0\right\} - dx_1(x_1 + x_2)$$

$$\frac{dx_1}{dt} = max\left\{\mu\left(1 - \frac{q_1}{Q}\right)x_{1,}0\right\} - dx_2(x_1 + x_2)$$

Implementing this change had some of the desired effect on the dynamics of the susceptible population. Figure S6 shows that, upon the implementation of these new death terms, the susceptible population was indeed seen to go extinct. This was, however, only a partial success. We would have preferred to see less oscillation in the resistant population, and for the susceptible population to fail over a longer period. Ideally, we want to see that PSA dynamics are driven by oscillations in the susceptible population, and that extinction of the susceptible population

coincided with a deviation between androgen and PSA behavior. This is open challenge that we may address in the future.

At the time, however, the primary problem was that by introducing interspecific competition we had significantly damaged the model's ability to reproduce androgen data. Therefore, we undertook one final modification in the hopes of improving upon that shortcoming: the introduction of a fifth differential equation.

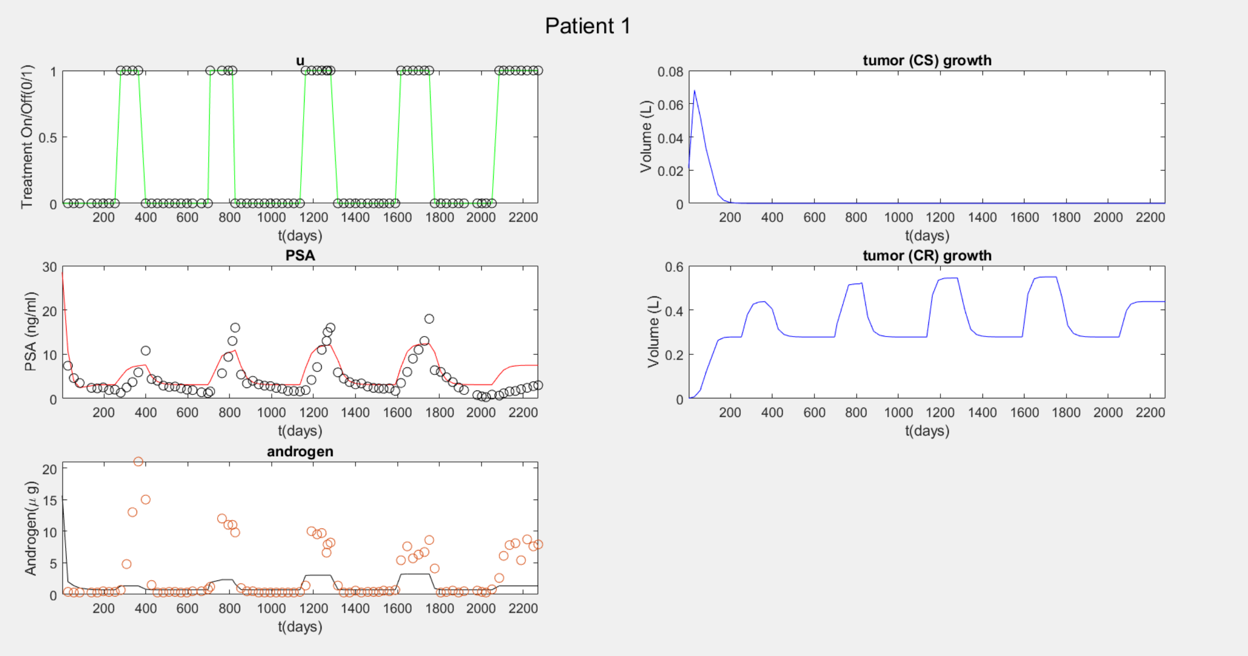

Figure S6: This figure shows the result of introducing interspecific competition. The susceptible subpopulation is now seen to die off, as expected. This result is only a partial success. The desired outcome was achieved, but likely too early, and too abruptly. We would also have preferred to see less oscillation in the resistant subpopulation. Finally, the ability of the model to reproduce androgen values has noticeably worsened.

The purpose of introducing a fifth differential equation was to compartmentalize the serum androgen, for which we had measurements, from the cell quota of androgen that motivated tumor growth and PSA production. We tested two different forms of this fifth differential equation. The first was published by Phan et al, and the second by Reckell et al (2,3). We found that, in the context of this model, the second equation produced better results.

Androgen equation (Phan et al):

$$\frac{dA}{dt} = \gamma_2 + \gamma_1(A_0 - A) - A_0\gamma_1\big(1 - u(t)\big)$$

Androgen equation (Reckell et al):

$$\frac{\mathrm{dA}}{\mathrm{dt}} = \gamma_2 + \gamma_1\left(1 - \frac{\mathrm{A}}{\mathrm{A}_0}\right)\mathrm{u(t)} - \delta\mathrm{A}$$

Implementing this fifth differential equation gives the final form of the model presented in the main body of the publication. Using this model, we were able to accomplish some of the goals we had when we began, but there remain opportunities for improvement. Upon introducing the fifth differential equation, we saw that the volumes of the resistant subpopulations again started to approach unrealistically large values. Furthermore, the model still struggles to reproduce androgen data for many of the patient datasets we tested. This is, to an extent, an expected consequence of how we weigh error (80% in favor of PSA), but we still believe that improvement is possible.

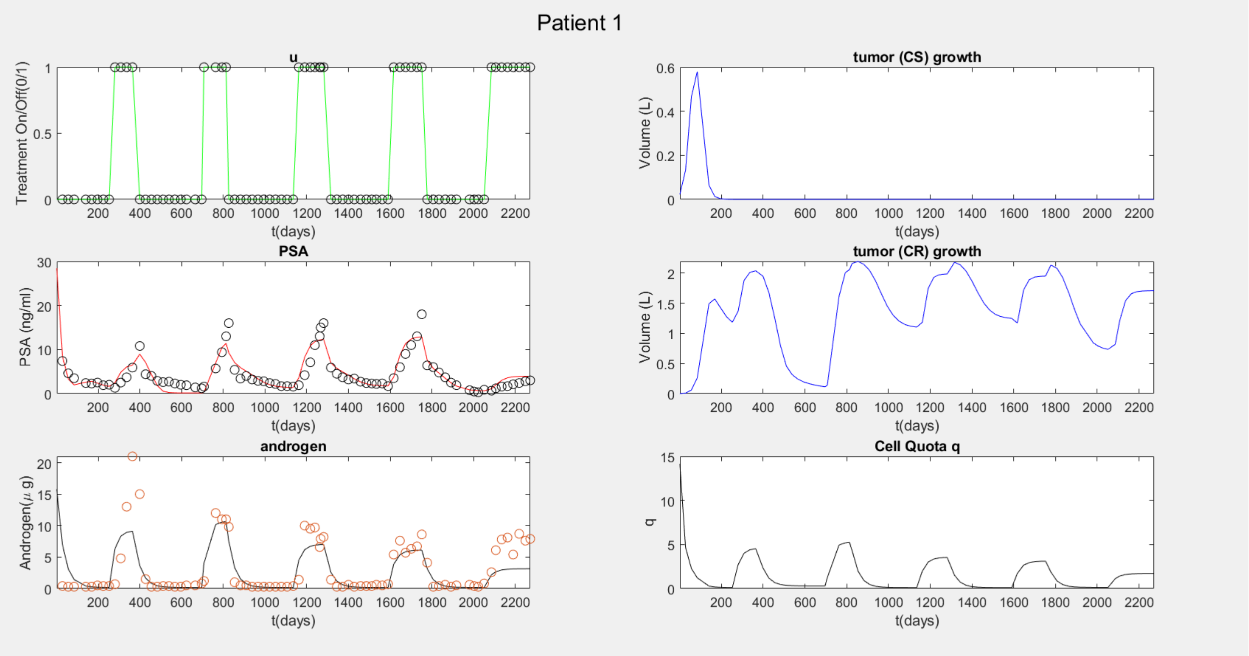

Figure S7: One result of compartmentalizing serum androgen from cell quota of androgen. The model's ability to recreate androgen has been improved, but the volumes of the resistant subpopulation have again grown to levels that may yet be too large. This is the final form of the model presented in this publication.

Objective Function:

Essential for the operation of the Matlab's fmincon function is the definition of the objective function, used to calculate the discrepancy between the model output and the actual data. We used the following objective function:

$$\sqrt{\frac{\sum_{i=1}^{N}\left(\frac{c_i - d_i}{\frac{c_i + d_i}{2}}\right)^2}{N}}$$

We chose to use this rather than something like sum of squared errors because we did not want the function to disproportionally weigh the largest errors. This reduced the power of outliers to disproportionally influence the optimized parameters. For the purposes of this investigation, PSA error was weighed far more heavily than androgen error (80% vs 20%).

Parameters and Fitting

The parameter values and ranges used in this model follow directly from work done by Phan et al (2).

Three of the fifteen parameters used in this model, *c*, *K*, and γ_2, are permanently fixed at some static value. We do this following the sensitivity analysis done by Phan et al, which led to them permanently fixing five of their parameters: *c*, *K*, δ_1, δ_2, and γ_2 (Note: Where they use δ for the density-dependent death rate, we use *d*) (2).

The mutation rate parameters *c* and *K* govern the rate that members of the susceptible population dynamically respond to treatment, and therefore move from the treatment susceptible to the treatment resistant population. The sensitivity analysis done by Phan et al demonstrated that these parameters were particularly insensitive, and that fixing these parameters was therefore a reliable way to improve the identifiability of the more critical parameters. A basis for the range of *c* was first published by Ideta et al (4). Following that, subsequent models, including this model, have used a fixed value of 0.00015 for *c*, and of 1 for *K* (1,2,5).

The next parameter γ_2 stands for the rate of secondary (adrenal) androgen production. This parameter is so insensitive that Phan et al set it to zero in their work (2). We did not set it to zero, but instead fixed it such that it would be a small percentage of γ_1, the primary androgen production parameter.

When it comes to the density death rate parameter *d* we deviate from the values used in earlier publications. The changes made to this model, and in particular the introduction of interspecific competition, require entirely new parameters. We therefore had no basis for setting a fixed value for *d* and allowed the parameter to be fitted by fmincon.

The twelve remaining parameters, including *d*, are all fitted in some way by fmincon. These parameters may be sorted into three different groups: four critical parameters, μ in a category by itself, and seven partially fitted parameters. The four parameters most sensitive and most important to our investigation are re-optimized for every half-cycle of treatment. In other words, every time the data indicates a switch in treatment status fmincon calculates a new best-fit value for that segment of treatment. This allows us to study the dynamics of these parameters over the whole course of treatment. μ is only fitted against the first half-cycle of treatment. The program runs for one half-cycle, discovers a set of best-fit parameters for that single half-cycle, and then fixes μ at that value. The seven remaining parameters are fitted against a two-cycle test segment, and then fixed at the resultant optimal value. In order, first the program runs the single half-cycle to find the fixed value of μ, then it runs for two whole cycles to find the fixed values of the seven partially-fitted parameters, and then the program runs the whole dataset, fitting and re-fitting only the four critical parameters.

The four sensitive, critical parameters are: q_2, representing the minimum amount of androgen required by the resistant cell population to survive and proliferate; γ_1, the primary (testicular) rate of androgen production; A_0, the maximum serum level of androgen; and σ_2, the rate of PSA production by the resistant cell population.

We cannot overstate the importance of the parameters q_2 and σ_2 to this investigation. It is q_2 that

is most responsible for simulating the development of resistance, and it forms a critical component of one of our proposed predictive indicators. Likewise, σ_2 is directly related to resistance, because it is a key driver of the divergence between androgen and PSA seen in patients as they become resistant to treatment. It is due to their direct effect on the modeling of resistance that these two parameters are considered critically important, and therefore re-fitted against every half-cycle of treatment.

We use the same upper and lower bounds for q_1 and q_2 as those published by Phan et al (2). They discovered work wherein was published the lowest and highest recorded serum androgen levels caused by hormonal therapy (0.41 nMol/L and 1.73 nMol/L) (6). We invariably see that androgen suppression therapy initially succeeds at reducing the volume of a tumor, so we may safely infer that the minimum cell quota for the susceptible population, q_1, lies somewhere in that range. Furthermore, we assume that q_2 is always less than q_1, and therefore set the upper bound of q_2 at the lower bound of q_1. There is no data to support the range of q_2, and so the lower bound is an estimate.

The maximum serum androgen, A_0, is one of the critical parameters because it is both patient-specific and especially sensitive. The data demonstrates that although values are typically below 27nm/mole, there is still considerable variation in each patient's maximum serum androgen measurement (7). For the purposes of this investigation, each patient's maximum measurement was identified and the upper and lower bounds of A_0, for that patient, were set to be ± 10 nMol from that maximum.

The parameter γ_1 has a singular impact on the shape and behavior of simulated androgen and PSA levels. Unfortunately, there is no known clinical value for the rate of testicular androgen production. This parameter is considered one our critical parameters both because of its sensitivity and because we have no basis from which to estimate its value. In their work, Phan et al started from a range published by Ideta et al, and then reduced that range until it suited their investigation (2,4). We follow their lead and use the same upper and lower bounds.

Regarding the remaining parameters: the sequence of modifications made in the formulation of our model have left us unable to use prior, published values for the density death rate parameter d. Therefore, we tested a range of estimated bounds, and selected the best performers. The bounds of q_1 are identical to the bounds of q_2, which have already been discussed. Likewise, the bounds for σ_1 are identical to the bounds for σ_2. Like d, we tested the upper and lower bounds of μ by testing a range of estimated values. In the end, however, we determined that the best performing bounds were the same as those published by Phan et al (2). Those bounds for μ were derived from work done by Berges et al, wherein they measured the proliferation rates of prostate cancer cell populations in both androgen-rich and androgen-poor environments (8). The work to establish the bounds of the remaining parameters: ϵ, b, m, and δ, was done by Baez et al, and Portz et al (1,5).

Trend of q_2

We examine the evolution of the cell quota parameter for the resistant cancer population, q_2. In theory, q_2 should decrease with each treatment, making the ratio q_1/q_n an increasing sequence where n is the current treatment cycle. We fit a linear model to this ratio for all patients.

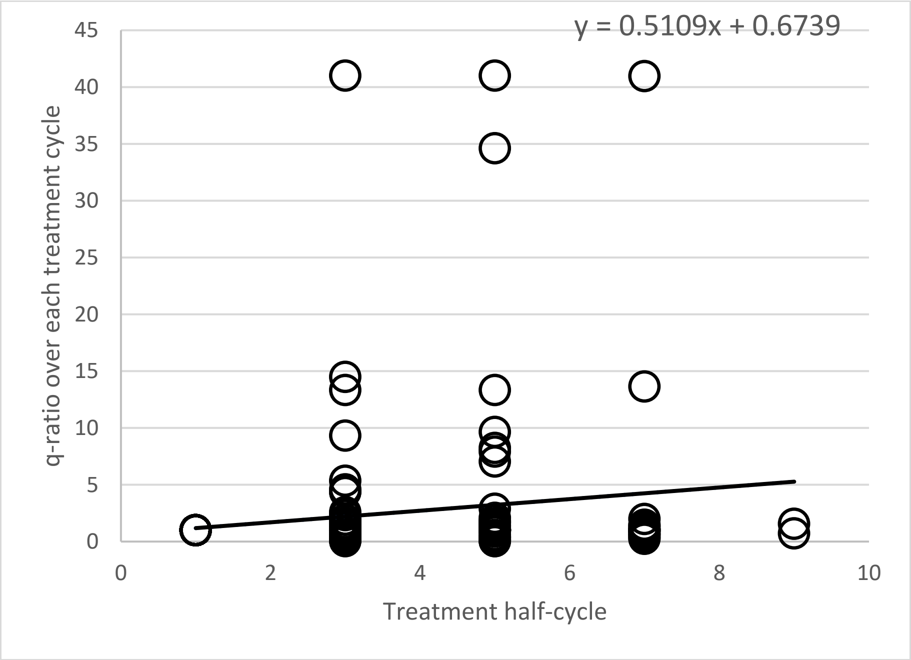

Figure S8: increasing trend of q_2 over each treatment cycle.

PSA and androgen summary statistics

We summarize the statistics regarding the recorded PSA and androgen data (Figure S9).

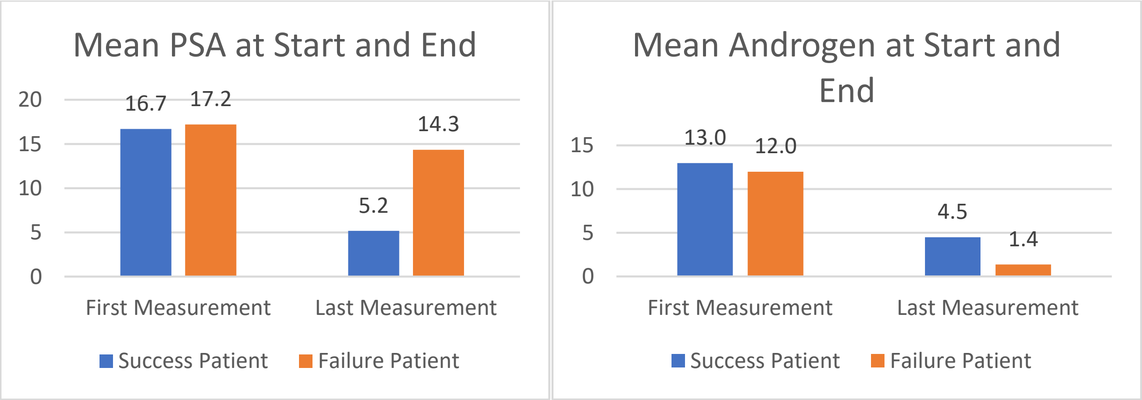

Figure S9: Summary statistics of PSA and androgen at the start and end of treatment.

Additionally, these are the more specific details regarding the data.

---Mean ---
First PSA data (success): 16.6941
First PSA data (failure): 17.195
Last PSA data (success): 5.171
Last PSA data (failure): 14.349
First androgen data (success): 12.9824
First androgen data (failure): 11.98
Last androgen data (success): 4.4784
Last androgen data (failure): 1.375

---Range ---
First PSA data (success): 60.8, [0.2, 61]
First PSA data (failure): 32.9, [7.9, 40.8]
Last PSA data (success): 42.68, [0.02, 42.7]
Last PSA data (failure): 57.94, [1.06, 59]
First androgen data (success): 21.2, [0.2, 21.4]
First androgen data (failure): 16.6, [4.8, 21.4]
Last androgen data (success): 18.4, [0.1, 18.5]
Last androgen data (failure): 5.6, [0.2, 5.8]

---Median ---
First PSA data (success): 14.1
First PSA data (failure): 14.7
Last PSA data (success): 3
Last PSA data (failure): 9.915
First androgen data (success): 13.2
First androgen data (failure): 12.2
Last androgen data (success): 3.2
Last androgen data (failure): 0.8

--- IQR ---
First PSA data (success): 11.55, [8.2, 19.75]
First PSA data (failure): 10.55, [10.95, 21.5]
Last PSA data (success): 8.125, [0.3575, 8.4825]
Last PSA data (failure): 8.75, [5.95, 14.7]
First androgen data (success): 6.25, [9.75, 16]
First androgen data (failure): 4.75, [8.55, 13.3]
Last androgen data (success): 6.3, [0.5, 6.8]
Last androgen data (failure): 1.15, [0.5, 1.65]

References

1. Baez J, Kuang Y. Mathematical models of androgen resistance in prostate cancer patients under intermittent androgen suppression therapy. Appl Sci 2016;6.

2. Phan T, Nguyen K, Sharma P, Kuang Y. The impact of intermittent androgen suppression therapy in prostate cancer modeling. Appl Sci 2018;9.

3. Reckell T, Nguyen K, Phan T, Crook S, Kostelich EJ, Kuang Y. Modeling the synergistic properties of drugs in hormonal treatment for prostate cancer. J Theor Biol 2021;514:110570.

4. Ideta AM, Tanaka G, Takeuchi T, Aihara K. A Mathematical Model of Intermittent Androgen Suppression for Prostate Cancer. J Nonlinear Sci 2008;18:593–614.

5. Portz T, Kuang Y, Nagy JD. A clinical data validated mathematical model of prostate cancer growth under intermittent androgen suppression therapy. AIP Adv 2012;2.

6. Nishiyama T. Serum testosterone levels after medical or surgical androgen deprivation: A comprehensive review of the literature. Urol Oncol: Semin Orig Investig 2014;32:38.e17-38.e28.

7. Bruchovsky N, Klotz L, Crook J, Malone S, Ludgate C, Morris WJ, et al. Final results of the Canadian prospective phase II trial of intermittent androgen suppression for men in biochemical recurrence after radiotherapy for locally advanced prostate cancer. Cancer 2006;107:389–95.

8. Berges RR, Vukanovic J, Epstein JI, CarMichel M, Cisek L, Johnson DE, et al. Implication of cell kinetic changes during the progression of human prostatic cancer. Clin Cancer Res 1995;1:473–80.
